# Endophytic Bacteria Isolated from *Panax ginseng* Improves Ginsenoside Accumulation in Adventitious Ginseng Root Culture

**DOI:** 10.3390/molecules22060837

**Published:** 2017-05-23

**Authors:** Xiaolin Song, Hao Wu, Zhenhao Yin, Meilan Lian, Chengri Yin

**Affiliations:** Key Laboratory of Natural Resources of Changbai Mountain and Functional Molecules, Ministry of Education, Yanbian University, Yanji 133002, China; xiaolinsong_1986@hotmail.com (X.S.); 2014001024@ybu.edu.cn (H.W.); yzh2015@ybu.edu.cn (Z.Y.)

**Keywords:** adventitious root culture, antitumor, endophytic bacterial elicitor, ginsenosides, *Panax ginseng*

## Abstract

Ginsenoside is the most important secondary metabolite of ginseng. Natural sources of wild ginseng have been overexploited. Although root culture could reduce the length of the growth cycle of ginseng, the number of ginsenosides is fewer and their contents are lower in adventitious roots of ginseng than that in ginseng cultivated in the field. In this study, we investigated the effects of endophytic bacterial elicitors on biomass and ginsenoside production in adventitious roots cultures of *Panax ginseng*. Endophyte LB 5-3 as an elicitor could increase biomass and ginsenoside accumulation in ginseng adventitious root culture. After 6 days elicitation with a 10.0 mL of strain LB 5-3, the content of total ginsenoside was 2.026 mg g^−1^ which was four times more than that in unchallenged roots. The combination of methyl jasmonate and strain LB 5-3 had a negative effect on ginseng adventitious root growth and ginsenoside production. The genomic DNA of strain LB 5-3 was sequenced, and was found to be most closely related to *Bacillus altitudinis* (KX230132.1). The challenged ginseng adventitious root extracts exerted inhibitory effect against the HepG2 cells, which IC_50_ value was 0.94 mg mL^−1^.

## 1. Introduction

*Panax ginseng* C.A. Meyer (Araliaceae) is one of the most famous and valuable oriental herbs. Ginsenoside is considered to be one of the most important secondary metabolism products in ginseng [1], and has various pharmacological and physiological effects including anticancer, antidiabetic, immunomodulatory, neuroprotective, radioprotective, antiamnestic and antistress properties [2,3].

In recent years, ginseng has been processed into many types of commercial health products. However, natural sources of wild ginseng have been overexploited. Current sources of ginseng mainly depend on field cultivation, which requires time and labor. Although root culture can shorten the growth cycle, reduce costs, the number of ginsenosides is fewer and their contents are lower in adventitious roots of ginseng than those in ginseng cultivated in the field, and this problem needs to be resolved urgently. In order to improve the biomass and ginsenoside production in ginseng adventitious root cultures, a number of studies have been carried out [4,5]. Due to the low content of ginsenoside in ginseng root culture, various elicitors have been developed to enhance the ginsenoside production. Most of the elicitors, such as jasmonic acid and methyl jasmonate (MeJA) can increase ginsenoside accumulation in ginseng root culture, but at the same time they reduce the biomass [5,6,7,8,9].

Endophytes are bacterial or fungal microorganisms that colonize healthy plant tissues without causing any apparent symptoms, and can protect their hosts by producing various substances [10]. Some studies have reported that endophytes as elicitors can stimulate secondary metabolite accumulation. Gao et al. found that foliar application combined with irrigation of ginseng plants with *Paenibacillus polymyxa* isolated from ginseng leaves enhanced plant growth parameters, reduced morbidity, and increased ginsenosides concentrations [11], however, this method requires the field-cultivated ginseng and thus field growth, which is time-consuming and costly. Wang et al. indicated that the extracts of *Colletotrichum* sp. which was isolated from *Artemisia annua* substantially improved the total concentrations of artemisinin in *Artemisia annua* hairy roots culture. And compared to the control, the maximum production of artemisinin increased 44% [12]. However, reports are lacking on the enhancement of ginsenoside production by live endophytic elicitors during *Panax* cell or organ cultures.

In this study, we challenged ginseng root cultures with live endophytic bacteria that were isolated from ginseng root. The advantages of this method are simple operation, and possible stimulation of the synthesis and accumulation of ginsenoside in ginseng adventitious root culture. To date, this is the first report on the use of a live endophyte as an elicitor for increasing ginsenoside production in ginseng adventitious root cultures.

## 2. Results and Discussion

### 2.1. Bacterial Endophytes as Elicitors

**Figure 1:**
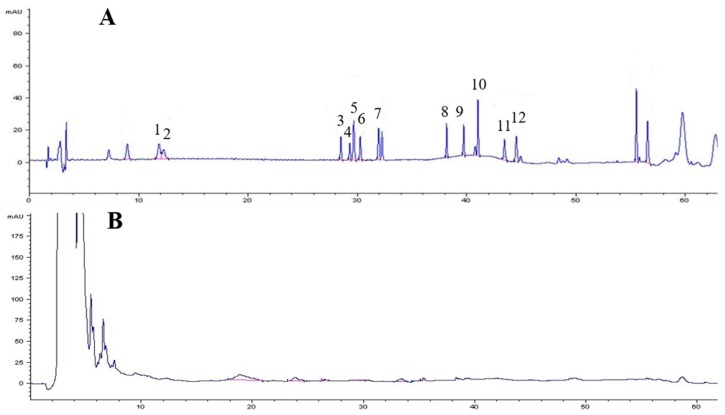
The HPLC profile of ginsenoside standards (**A**); and the fermentation broth of strain LB 5-3 (**B**). The peaks 1–12 represents ginsenosides Rg1, Re, Rh1, Rb1, F1, Rb2, Rd, F2, Rg3, CK, Rh2, and PPD respectively.

A total of 39 species of endophytes were isolated from field-cultivated ginseng roots. Of these isolates, 29 species of endophytes did not produce ginsenosides. All 29 species were used to screen elicitors for the induction experiment; none of these species increased biomass or improved ginsenoside accumulation in adventitious root culture except strain LB 5-3, thus strain LB 5-3 was chosen as the elicitor for the elicitation experiment. Strain LB 5-3 was cultivated and incubated on LB agar medium for 7 days at 30 °C and 150 rpm. Following incubation, the fermentation broth was extracted with *n*-BuOH three times and combined with the supernatant. The supernatant was concentrated in vacuum to dryness, the residue was then dissolved in 1.0 mL of HPLC grade methanol, and filtered through a 0.22-μm membrane filter prior to analysis of the production of ginsenosides using HPLC, and the results are shown in Figure 1. Strain LB 5-3 did not produce any ginsenosides.

**Figure 2:**
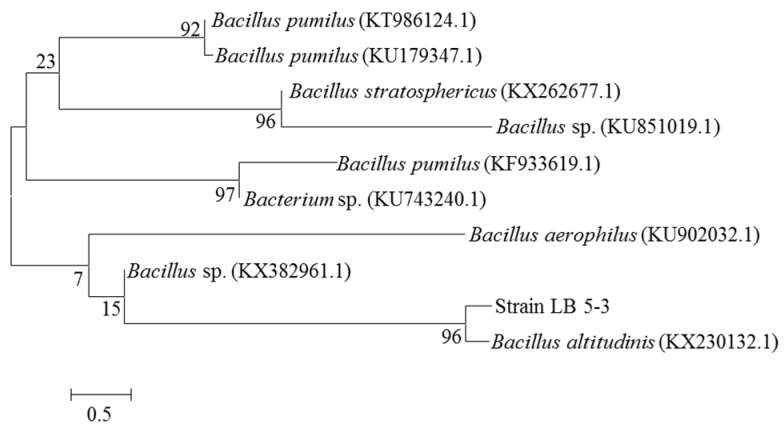
Phylogenetic tree based on 16S rDNA gene sequences showing the phylogenetic relationships between endophyte LB 5-3 and related species.

**Figure 3:**
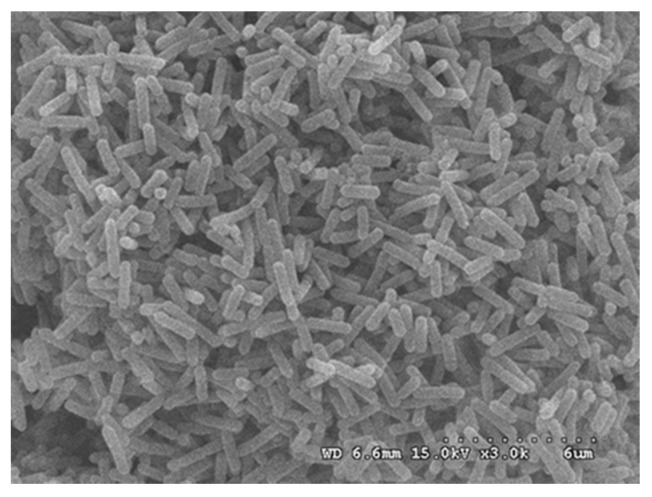
The SEM image of LB 5-3 on LB agar medium after 20 h of culture.

The genomic DNA of endophyte LB 5-3 was sequenced and compared to the GenBank databases. Phylogenetic analysis based on 16S rDNA gene sequences indicated that strain LB 5-3 belonged to the genus *Bacillus* and was most closely related to *Bacillus altitudinis* (KX230132.1); these results are shown in Figure 2. Endophyte LB 5-3 was observed by SEM. Figure 3 shows the SEM image of colonies of endophyte LB 5-3 on LB agar medium after 20 h of culture, and shows that endophyte LB 5-3 is a typical rod-shaped bacteria, with a with a length of approximately 1–2 μm; it does not have a flagellum or capsule and the surface and the edge of the strain is smooth and clear.

### 2.2. Effect of the Endophytic Elicitor on Biomass and Ginsenoside Production

To analyze the effect of inoculation concentrations of strain LB 5-3 suspension on the enhancement of root growth and the ginsenoside contents in adventitious root cultures, ginseng adventitious roots that were cultured in the bioreactor for 30 days were transferred into flasks. For flask cultures, 5.0 g fresh roots were cultured for 12 days with different inoculation volumes (0, 1.0, 2.5, 5.0, 10.0, 15.0, 20.0, 25.0) of endophyte LB 5-3 suspension. The flask without endophyte suspension acted as the control. Root growth (fresh weight, dry weight) and ginsenoside content were measured after 12 days.

**Figure 4:**
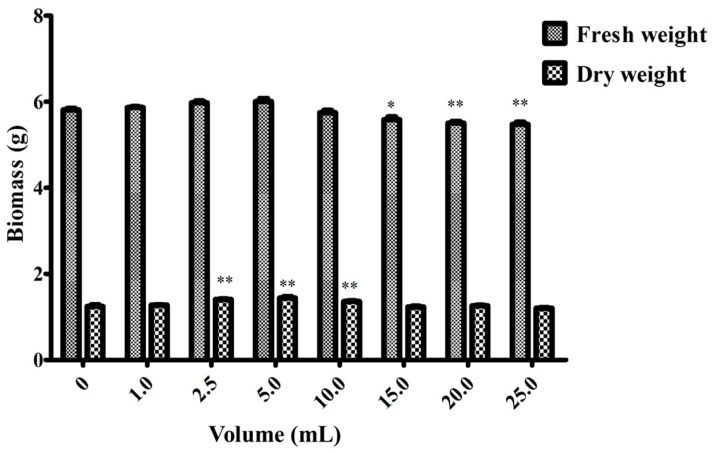
Effects of different endophyte suspension inoculation volumes on biomass after 12 days of flask culture. Data represent the mean ± standard deviation of three replicates. Data represent the mean ± standard deviation of three replicates. * Significantly different values between the treatment and control groups at *p* < 0.05. ** Very significantly different values between the treatment and control groups at *p* < 0.01.

**Figure 5:**
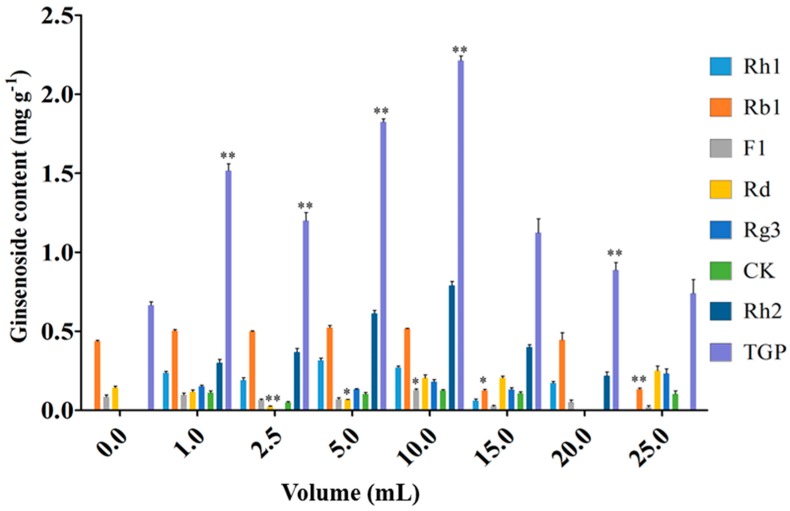
Effects of different endophyte suspension inoculation volumes on ginsenosides biosynthesis after 12 days of flask culture. TGP represents the total ginsenoside production. Data represents the mean ± standard deviation of three replicates. Data represent the mean ± standard deviation of three replicates. * Significantly different values between the treatment and control groups at *p* < 0.05. ** Very significantly different values between the treatment and control groups at *p* < 0.01.

Figure 4 and Figure 5 show the effects of endophyte suspension inoculation volume on ginseng adventitious root growth and ginsenoside production. The results showed that an appropriate inoculation volume of endophyte suspension enhanced root growth and ginsenoside contents. When the inoculation volume of strain LB 5-3 suspension ranged from 0 to 5.0 mL, the fresh weight and dry weight of ginseng adventitious roots increased with increasing inoculation volume and peaked at 5.0 mL. As a result, 6.010 g of fresh weight and 1.441 g of dry weight were obtained. When the inoculation volume ranged from 10.0 to 25.0 mL, the fresh weight and dry weight of the roots decreased with increasing inoculation volume. Ginsenoside content was significantly enhanced by the addition of strain LB 5-3 suspension. Total ginsenoside content reached a highest level at an inoculation volume of 10.0 mL. When the suspension inoculation volume ranged from 15.0 to 25.0 mL, the ginsenoside content in the roots decreased, which may have been due to insufficient nutrients in the broth for metabolism, and the bacteria metabolized ginsenosides as nutrients [13]. Due to the high level of ginsenoside accumulation in ginseng adventitious roots, 10.0 mL of LB 5-3 was considered the optimal inoculation volume.

To investigate the effect of different elicitation times on the enhancement of root growth and the contents of ginsenoside in ginseng adventitious root cultures, 5.0 g fresh ginseng adventitious roots that cultured in the bioreactor for 30 days were subcultured into a 150-mL flask containing MS medium. The inoculation volume of strain LB 5-3 was 10.0 mL. The elicited cultures were harvested after different cultivation times. The flask without endophyte suspension was acted as the control. Root growth (fresh weight, dry weight) and ginsenoside content were analyzed. When the induction time was increased to 6 days, the ginsenoside content was significantly increased and the content reached 2.026 mg g^−1^ following the addition of strain LB 5-3 suspension, which was four times that of the control. Due to the high level of ginsenoside accumulation in ginseng adventitious roots, 6 days was considered the optimal inoculation time.

### 2.3. Effect of MeJA, Endophytic Bacteria and Their Combination during Flask Culture

**Figure 6:**
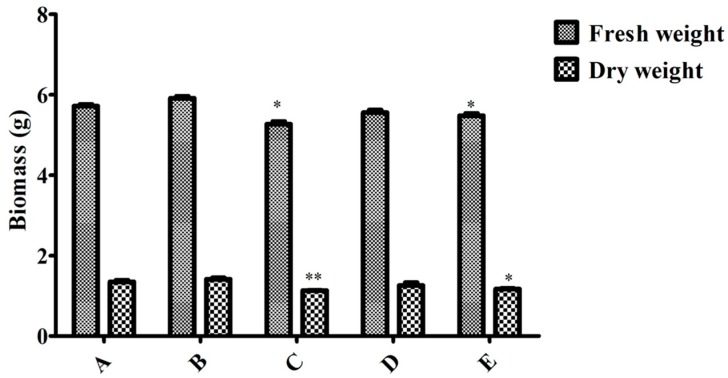
Effect of MeJA, endophytic bacteria and their combination on biomass in flask culture. A, B, C, D, and E represent the different additions of endophytic bacteria and MeJA. A = without endophytic bacteria or MeJA treatment, B = 10.0 mL endophyte suspension, C = 100 μM MeJA, D = 5.0 mL endophyte suspension + 50 μM MeJA, and E = 10.0 mL endophyte suspension + 100 μM MeJA. Data represent the mean ± standard deviation of three replicates. * Significantly different values between the treatment and control groups at *p* < 0.05. ** Very significantly different values between the treatment and control groups at *p* < 0.01.

**Figure 7:**
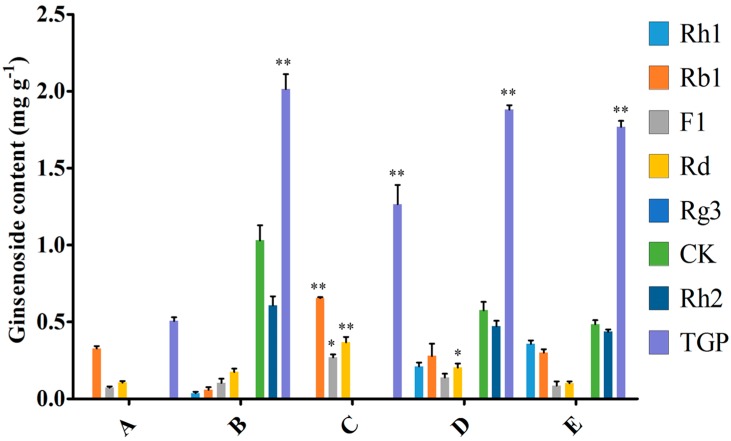
Effect of MeJA, endophytic bacteria and their combination on ginsenosides biosynthesis in flask culture. A, B, C, D, E represents the different addition method of endophytic bacteria and MeJA. A = without endophytic bacteria or MeJA treatment, B = 10.0 mL endophyte suspension, C = 100 μM MeJA, D = 5.0 mL endophyte suspension + 50 μM MeJA, and E =10.0 mL endophyte suspension + 100 μM MeJA. TGP represents the total ginsenoside production. Data represent the mean ± standard deviation of three replicates. * Significantly different values between the treatment and control groups at *p* < 0.05. ** Very significantly different values between the treatment and control groups at *p* < 0.01.

To compare induction with MeJA and the endophytic bacteria elicitor on adventitious root cultures, the optimal inoculation volume of endophyte LB 5-3 and MeJA were selected and five treatments (control, 10.0 mL endophyte suspension, 100 μM MeJA, 5.0 mL endophyte suspension + 50 μM MeJA, and 10.0 mL endophyte suspension + 100 μM MeJA) were designed, and the control was not treated with MeJA or endophytic bacteria elicitor. After 6 days of cultivation, the biomass and ginsenoside contents were determined. Figure 6 indicates the effects of the different additions of LB 5-3 and MeJA on ginseng adventitious root growth in flask culture. As shown in Figure 6, fresh weight and dry weight of the roots treated with 10.0 mL LB 5-3 suspension were 5.914 g and 1.415 g, respectively, which were higher than those obtained with the other treatments. The biomass of roots treated with 100 μM MeJA was lowest. The fresh weight and dry weight of the roots treated with 5.0 mL endophyte suspension + 50 μM MeJA, and 10.0 mL endophyte suspension + 100 μM MeJA were higher than the 100 μM MeJA group but lower than the control group. Figure 7 shows the effects of the different additions of LB 5-3 and MeJA on ginsenoside production in the roots in flask culture. The results showed that MeJA can be used to enhance the yield of ginsenoside. Ginsenoside production in the roots treated with 10.0 mL LB 5-3 suspension was 2.036 mg g^−1^ which was much higher than that obtained with the other treatments. Ginsenoside production in the control group was the lowest. Ginsenoside production in the roots treated with 5.0 mL endophyte suspension + 50 μM MeJA, and 10.0 mL endophyte suspension + 100 μM MeJA were higher than that in the 100 μM MeJA group and the control group.

Due to the low content of secondary metabolites in root culture, various strategies have been employed to increase the yield of secondary metabolites including modulation of culture media or environmental conditions and the addition of precursors and elicitors [14,15,16,17,18]. Among these strategies, elicitation is the most efficient method for the production of secondary metabolites. Elicitor can improve the contents of ginsenosides; especially, different types, timings, and duration of elicitation, which can affect ginsenoside production and heterogeneity [19]. Many studies have reported that MeJA can enhance the production of ginsenosides. However, the biomass in plant cell, tissue, and organ culture systems decreased after MeJA treatment [6,20,21,22,23]. This may be due to the direct toxic effect of MeJA [23]. For example, Kim et al. found that total ginsenosides in ginseng adventitious roots increased after elicitation with 100 μM MeJA, but the dry weight decreased after MeJA treatment [21]. Therefore, the degree of culture biomass inhibition should be considered before choosing an elicitor. Some studies found that the combination of elicitors increased the accumulation of secondary metabolites, such as 100 mg mL^−1^ lactoalbumin hydrolysate mixed with 2 mg L^−1^ MeJA, which enhanced ginsenoside accumulation in *Panax quinquefolius* cells [24]. To compare elicitation by MeJA and strain LB 5-3 on adventitious root cultures, MeJA, endophytic bacteria and their combination were added during flask culture. Figure 7 shows that MeJA can enhance the yield of total ginsenoside, but the combination of MeJA and strain LB 5-3 had a negative effect of ginseng adventitious root growth and ginsenoside production compared with 10.0 mL strain LB 5-3 suspension. This is possibly because the toxic effect of MeJA inhibited the growth of ginseng adventitious roots and strain LB 5-3 [23]. Huang et al. found that the biomass of *Panax quinquefolium* L. cells cultures decreased after elicitation with 2 mg L^−1^ MeJA, but the ginsenoside content improved and was 1.86 times higher than the control group [24]. Thanh et al. also found ginsenoside accumulation in ginseng cell cultures was enhanced by elicitation of 200 μM MJ, and the ginsenoside content was two times that of the control [25]. In the present study, live endophyte not only increased biomass, but also improved ginsenoside accumulation in adventitious root culture, the ginsenoside production was four times that of the control, which was higher than other studies which used MeJA as an elicitor [24,25].

Moreover, in Lian’s research, 30-day-old ginseng adventitious roots in bioreactors were treated with extracts of *Alternaria panax* (4.0 mg L^−1^) for 8 days, and the maximum ginsenoside production was twice that of the control group, but the biomass of roots was reduced [26]. In this study, ginseng adventitious root culture was challenged with live endophyte, the biomass was increased, the ginsenoside content was improved which was four times that of the control. Therefore, the live endophyte LB 5-3 could be a potential elicitor for the production of ginsenosides.

### 2.4. In Vitro Anticancer Activity Assay

**Figure 8:**
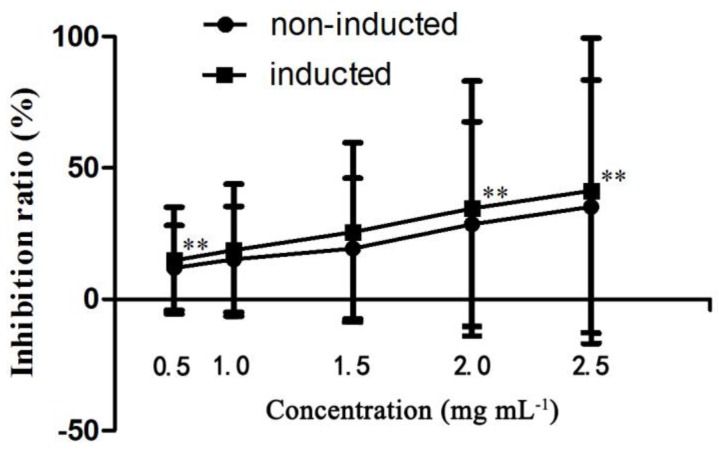
The antiproliferative effects of inducted ginseng adventitious roots on HepG2 cells. Data represents the mean ± standard deviation of three replicates. ** The very significantly different values between same content of inducted and un-inducted groups at *p* < 0.01.

Using the human liver cancer cell line HepG2, the antiproliferative effects of induced ginseng adventitious root extracts at a concentration range of 0.5–2.0 mg mL^−1^ were evaluated and compared. As shown in Figure 8, the anticancer effect of the induced ginseng adventitious root extracts was stronger compared with non-induced ginseng adventitious roots at the same concentrations. The extracts of ginseng adventitious roots induced by strain LB 5-3 had an inhibitory effect on HepG2 liver cancer cells, leading to significant decreases in cell viability. The IC_50_ value of the induced ginseng adventitious root extracts was 0.94 mg mL^−1^, lower than that of non-induced ginseng adventitious root extracts, which was 1.30 mg mL^−1^. Some studies were found that CK and Rh2 have strong anticancer activity, even very low content of CK (20 µM) and Rh2 (10 µM) have strong cytotoxic effect on HepG2 [27,28]. In Figure 8, the anticancer effect of the inducted ginseng adventitious root extracts was stronger than the same concentration of non-inducted root extracts, the biological activities might be the result of the higher content of ginsenoside CK, Rh2 in inducted ginseng adventitious root extracts.

## 3. Materials and Methods

### 3.1. Chemicals

Standard ginsenosides (Rg1, Re, Rh1, Rb1, F1, Rb2, Rd, F2, Rg3, CK, Rh2 and PPD) were purchased from the Shanghai Winherb Medical Technology Co., Ltd. (Shanghai, China). Silica gel-60 was obtained from Merck KGaA (Darmstadt, Germany). The bacterial DNA extraction kit, PCR purification kit and API ZYM kit were obtained from Bioneer Corporation (Daejeon, Korea). MeJA, 2,4-dichlorophenoxyacetic acid (2,4-D), and indole-3-butytric (IBA) were obtained from Sigma Chemical Co. (St. Louis, MO, USA).

### 3.2. Isolation of Endophytes in Panax ginseng Roots

Ginseng samples were obtained from a ginseng field in Yanji, China. The ginseng was kept in a refrigerator at 4 °C, and endophytes were isolated within 48 h. The ginseng was thoroughly washed, cleaned of soil, and then surface-disinfected four times with the following sequence of immersions: 1 min in 70% (*v*/*v*) ethanol, 5–10 min in 5% NaClO, 1 min in 70% ethanol, and 1 min in sterile water. The ginseng epidermis was removed with a sterile scalpel. Segments (0.5 cm × 0.5 cm) were cut and pressed onto a potato dextrose agar plate, R2A agar plate and Lysogeny Broth (LB) agar plate medium (containing tryptone 10.0 g, yeast extract 5.0 g, agar 10 g and NaCl 10.0 g L^−1^) and incubated at 30 °C for 5–7 days. Single colonies from these plates were purified by transfer onto new plates.

### 3.3. Screening Bacterial Endophytes as Elicitors

Endophytes were cultivated and incubated on appropriate liquid medium (solid medium cultivated microorganism without agar) for 7 days at 30 °C and 150 rpm. Following incubation, the fermentation broth was extracted with aqueous saturated *n*-butyl alcohol (*n*-BuOH) three times and combined with the supernatant. The supernatant was concentrated in vacuum to dryness, and the residue was then dissolved in methanol (HPLC grade), and filtered through a 0.22-μm membrane filter prior to analysis of the production of ginsenosides using HPLC. The retention time of the product was compared with 12 ginsenoside standards, and the endophytes that did not produce ginsenosides were chosen as microbial elicitors for ginsenoside production in ginseng root culture.

### 3.4. Cultivation of Ginseng Adventitious Roots in an Air-Lift Bioreactor

Ginseng samples were obtained from a ginseng field in Yanji, China, washed and blotted dry, then surface-disinfected with 75% (*v*/*v*) ethanol for 5 min. The ginseng epidermis was removed with a sterile scalpel. After that, the internal tissue of ginseng was cut into 1.0 cm × 1.0 cm sections and inoculated onto MS media [29] which contained 1.0 mg L^−1^ 2,4-D, 30 g L^−1^ sucrose and 6.5 g L^−1^ agar. The callus was induced at 25 °C in the dark for 30 days.

The callus was then inoculated into MS medium (pH 5.8, containing 5 mg L^−1^ IBA, and 30 g L^−1^ sucrose) for the induction of adventitious roots, and cultured at 25 °C in the dark for 30 days. Following cultivation, the ginseng adventitious roots were subcultured under the same conditions.

The ginseng adventitious roots were cultured in a 5-L air-lift bioreactor containing 4 L MS medium (pH 5.8, 5 mg L^−1^ IBA, 30 g L^−1^ sucrose), and the inoculum was 20 g L^−1^ fresh weight of excised ginseng adventitious roots at 25 °C in the dark. Filtered air was provided during cultivation and the airflow rate was 100 mL min^−1^. The composition of supplied air was the same as atmospheric air.

### 3.5. Endophytic Bacteria Challenge Experiment

Ginseng adventitious roots that were cultured in the bioreactor for 30 days were transferred into a 150-mL flask. Five grams fresh roots were inoculated into the flask containing MS medium (pH 5.8, 5 mg L^−1^ IBA, 30 g L^−1^ sucrose). For the bacterial elicitation experiment, the endophytic bacteria were inoculated into MS medium and kept at 30 °C, 150 rpm, then diluted to OD_600_ = 0.5 (5 × 10^7^ cfu mL^−1^) [30], and subsequently added to the flask containing 5.0 g fresh roots and medium. Following incubation, the ginseng adventitious roots were washed with distilled water and blotted dry, the roots were then measured and the weight was recorded. Dry weight of the ginseng adventitious roots was measured after drying in an oven. The flask without elicitor acted as the control.

### 3.6. Optimization of the Elicitation Conditions

To optimize induction of the bacterial suspension on adventitious root cultures, the inoculation volume of strain LB 5-3 was adjusted to 1.0–25.0 mL (the total volume of medium in the flask was 100.0 mL), solutions were at final concentrations of approximately 5.0 × 10^5^, 1.25 × 10^6^, 2.5 × 10^6^, 5 × 10^6^, 7.5 × 10^6^, 1.0 × 10^7^, and 1.25 × 10^7^ cfu mL^−1^, and the induction time (0–12 days) after injection of the elicitor was examined.

### 3.7. Effect of MeJA, Endophytic Bacteria and Their Combination during Flask Culture

MeJA is considered to be a signaling molecule under biotic and abiotic stresses, and is an effective elicitor widely used in plant cultures [20]. To compare the elicitation of MeJA and endophytic bacteria elicitor on adventitious root cultures, the optimal concentration of endophytic bacteria elicitor and MeJA were selected and five treatments were designed. The control was not treated with MeJA or endophytic bacteria elicitor. After 6 days of cultivation, biomass and ginsenoside accumulation were determined.

### 3.8. Cell Survival Assay

We evaluated the effect of induced ginseng adventitious root extracts on the survival of HepG2 cells (human hepatoma cell line). The ginseng adventitious root powders that were cultured under optimized elicitation conditions were extracted with the same volume of methanol. The liquid fraction was concentrated in vacuum to dryness, and the residue was then dissolved in distilled water. After defatting with ethyl ether, the aqueous layer was further extracted with aqueous saturated *n*-BuOH three times and combined with the supernatant. The supernatant was concentrated in vacuum to dryness to obtain the root extracts.

HepG2 cells were cultured in Dulbecco’s modified Eagle’s medium (DMEM) supplemented with 10% (*v*/*v*) fetal bovine serum (Gibco, Waltham, MA, USA) at a temperature of 37 °C, 5% CO_2_ concentration and 95% relative humidity. HepG2 cells were seeded at 5 × 10^3^ cells mL^−1^ in 96-well plates (180 μL per well). The root extracts were dissolved in dimethyl sulfoxide (DMSO) and filtered through a 0.22-μm membrane filter. Each sample was prepared in triplicate, and 20 μL was added to each well. The final concentrations were 0.25, 0.5, 1.0, 1.5 and 2.0 mg mL^−1^. These cells were incubated for 48 h. Then 20 μL 3-(4,5-dimethyl-2-thiazolyl)-2,5-diphenyl-2-*H*-tetrazolium bromide (MTT, 5 mg mL^−1^ in PBS) were added to each well and incubated for 4 h. The supernatant was removed and the formed formazan crystals were dissolved with 150 μL DMSO. Each plate was shaken for 20 min and the OD values were read at 492 nm on a microplate reader within 30 min. The IC_50_ value was defined as the concentration of sample needed to reduce 50% of absorbance relative to the vehicle-treated control.

### 3.9. Scanning Electron Microscopy (SEM)

For SEM analyses, the strain LB 5-3 was plated onto LB medium and cultured at 37 °C for 20 h. The colony of strain LB 5-3 taken from the LB medium was fixed with glutaraldehyde 2.5% *v*/*v* in a 0.1 M phosphate buffer solution (PBS, pH 7.2), for 24 h at 4 °C. The samples were then dehydrated by 10%, 30%, 50%, 70%, 85%, 95% and 100% of ethanol, and the dehydration time for each alcoholic solution was 15 min except the higher concentrations. Finally, metallization of the dehydrated strain LB 5-3 was carried out and observed using a Hitachi S-450 SEM (Hitachi, Ltd., Tokyo, Japan), at 15.0 kV on a JEOL JMS 6480 LV computer (Scanning Electron Microscopy and Microanalysis by X-ray Scattering; JEOL, Tokyo, Japan).

### 3.10. Analytical Methods

Dried ginseng adventitious root powders (0.5 g) were extracted with 1.0 mL methyl alcohol three times and combined with the liquid fraction. The liquid fraction was concentrated in vacuum to dryness, and the residue was then dissolved in distilled water. After defatting with ethyl ether, the aqueous layer was further extracted with aqueous saturated *n*-BuOH three times and combined with the supernatant. The supernatant was concentrated in vacuum to dryness, and the residue was then dissolved in methanol (HPLC grade), filtered through a 0.22-μm membrane filter and analyzed by HPLC.

HPLC analysis was performed using BDS-HYPERSIL-ODS C18 columns (4.6 × 250 mm, ID 5 μm) connected to an Agilent 1100 HPLC system (Agilent Technologies, Santa Clara, CA, USA). The elution phase was H_2_O (A) and CH_3_CN (B), and gradient elution was started with 77% solvent A and 23% solvent B and was then changed to: B from 23% to 46%, 0–13 min; B from 46% to 68%, 13–33 min; B from 68% to 100%, 45–55 min; B from 100% to 23%, 60–63 min. The flow rate was 1.0 mL min^−1^, and the samples were detected by absorption at 203 nm. The injection volume was 10.0 µL.

### 3.11. Standard Curve Preparation

For linearity validation and standard curve preparation, the standards of Rg3, Rh1, Rb1, Rd, F1, CK and Rh2 were accurately weighed and dissolved with methanol (HPLC grade) to obtain a stock solution at a series of concentrations. The different concentrations of ginsenoside standards were filtered and then analyzed by HPLC. The peak area of each ginsenoside standard was recorded and the standard curve was calculated according to the peak area and concentrations. The data were submitted to linear regression analysis in Microsoft Excel software (Microsoft Corporation, Redmond, WA, USA) and the regression equation for each ginsenoside is shown in Table 1.

### 3.12. Phylogenetic Analysis

The genomic DNA of strain LB 5-3 was extracted using the bacterial DNA extraction kit. The DNA quality was assessed using 1 × TAE agarose gel electrophoresis stained with ethidium bromide (EB) nucleic acid gel stain. The bacterial 16S rDNA was amplified using universal primers 27F (5′-AGAGTTTGATCMTGGCTCAG-3′) and 1492R (5′-TACGGYTACCTTGTTACGACTT-3′). After the PCR process, the PCR product was detected by the Beijing Genewiz Biotechnology Co., Ltd. (Beijing, China). The sequence of strain LB 5-3 was compared to the GenBank databases using the BLAST algorithm. A phylogenetic tree was constructed using the neighbor-joining method and the MEGA 6.0 program with bootstrap values based on 1000 replicates.

### 3.13. Statistical Analysis

The biomass and ginsenoside contents were expressed as mean ± standard deviation (SD). Statistical analyses were carried out using GraphPad Prism software (GraphPad, San Diego, CA, USA).

## 4. Conclusions

This research demonstrates that the endophytic bacterium, *Bacillus stratosphericus*, is an effective elicitor and can increase ginseng adventitious root growth and ginsenoside concentration. Live microbial cultures showed strong elicitation in terms of ginseng production. In conclusion, the root cultured together with the live microorganism may be a useful system for the production of secondary metabolites.

## Figures and Tables

**Table 1:** The standard curve of ginsenosides, *x* represents the concentration of ginsenoside, *y* represents the peak area of ginsenoside.

Ginsenoside	Linear Range (mg mL^−1^)	Linear Equation	Correlation Coefficient (r2)
Rh1	0.002–2.000	*y* = 2429.3*x* + 21.901	0.999
Rb1	0.004–4.000	*y* = 1598.8*x* + 35.703	0.9988
F1	0.004–4.000	*y* = 2630.8*x* + 10.136	0.9992
Rd	0.004–4.000	*y* = 1604.2*x* + 20.314	0.9989
Rg3	0.002–2.000	*y* = 2064.8*x* + 42.395	0.9987
CK	0.002–1.500	*y* = 2961.7*x* + 12.920	0.9992
Rh2	0.002–1.500	*y* = 2945.2*x* + 42.138	0.999

## Supplementary Materials

Supplementary materials are available online.
