# Supplementary material for: Endophytic Bacteria Isolated from Panax ginseng Improves Ginsenoside Accumulation in Adventitious Ginseng Root Culture
Source: Molecules. 2017 May 23;22(6):837. doi: 10.3390/molecules22060837 (PMC6152624; doi:10.3390/molecules22060837)
Supplement: Supplementary file 1 [file molecules-22-00837-s001.pdf]

Endophytic Bacteria Isolated from *Panax ginseng* Improves Ginsenoside Accumulation in Adventitious Ginseng Root Culture

Xiaolin Song, Hao Wu, Zhenhao Yin, Meilan Lian * and Chengri Yin *

Key Laboratory of Natural Resources of Changbai Mountain and Functional Molecules, Ministry of Education, Yanbian University, Yanji 133002, China; xiaolinsong_1986@hotmail.com (X.S.); 2014001024@ybu.edu.cn (H.W.); yzh2015@ybu.edu.cn (Z.Y.)

* Correspondence: mllian@ybu.edu.cn (M.L.); yin_cheng_ri@hotmail.com (C.Y.); Tel.: +86-433-2435625 (M.L.); +86-433-2732042 (C.Y.); Fax: +86-433-2732207 (C.Y.)

LB 5-3 16S rDNA gene sequences

>LB 5-3

TGCTATACATGCAGTCGAGCGGACAGAAGGGAGCTTGCTCCCGGATGTTAG

CGGCGGACGGGTGAGTAACACGTGGGTAACCTGCCTGTAAGACTGGGATA

ACTCCGGGAAACCGGAGCTAATACCGGATAGTTCCTTGAACCGCATGGTTC

AAGGATGAAAGACGGTTTCGGCTGTCACTTACAGATGGACCCGCGGCGCA

TTAGCTAGTTGGTGAGGTAACGGCTCACCAAGGCGACGATGCGTAGCCGA

CCTGAGAGGGTGATCGGCCACACTGGGACTGAGACACGGCCCAGACTCCT

ACGGGAGGCAGCAGTAGGGAATCTTCCGCAATGGACGAAAGTCTGACGGA

GCAACGCCGCGTGAGTGATGAAGGTTTTCGGATCGTAAAGCTCTGTTGTTA

GGGAAGAACAAGTGCAAGAGTAACTGCTTGCACCTTGACGGTACCTAACC

AGAAAGCCACGGCTAACTACGTGCCAGCAGCCGCGGTAATACGTAGGTGG

CAAGCGTTGTCCGGAATTATTGGGCGTAAAGGGCTCGCAGGCGGTTTCTTA

AGTCTGATGTGAAAGCCCCCGGCTCAACCGGGGAGGGTCATTGGAAACTG

GGAAACTTGAGTGCAGAAGAGGAGAGTGGAATTCCACGTGTAGCGGTGAA

ATGCGTAGAGATGTGGAGGAACACCAGTGGCGAAGGCGACTCTCTGGTCT

GTAACTGACGCTGAGGAGCGAAAGCGTGGGGAGCGAACAGGATTAGATAC

CCTGGTAGTCCACGCCGTAAACGATGAGTGCTAAGTGTTAGGGGGTTTCCG

CCCCTTAGTGCTGCAGCTAACGCATTAAGCACTCCGCCTGGGGAGTACGGT

CGCAAGACTGAAACTCAAAGGAATTGACGGGGGCCCGCACAAG

CGGTGGAGCATGTGGTTTAATTCGAAGCAACGCGAAGAACCTTACCAGGT

CTTGACATCCTCTGACAACCCTAGAGATAGGGCTTTCCCTTCGGGGACAGA

GTGACAGGTGGTGCATGGTTGTCGTCAGCTCGTGTCGTGAGATGTTGGGTT

AAGTCCCGCAACGAGCGCAACCCTTGATCTTAGTTGCCAGCATTCAGTTGG

GCACTCTAAGGTGACTGCCGGTGACAAACCGGAGGAAGGTGGGGATGACG

TCAAATCATCATGCCCCTTATGACCTGGGCTACACACGTGCTACAATGGACA

GAACAAAGGGCTGCGAGACCGCAAGGTTTAGCCAATCCCACAAATCTGTT

CTCAGTTCGGATCGCAGTCTGCAACTCGACTGCGTGAAGCTGGAATCGCTA

GTAATCGCGGATCAGCATGCCGCGGTGAATACGTTCCCGGGCCTTGTACAC

ACCGCCCGTCACACCACGAGAGTTTGCAACACCCGAAGTCGGTGAGGTAA

CCTTTAGGAGCCAGCCGCCGAAGGTGGGCAAGGATAGG
